# Evaluation of CD3+CD56+ NKT and CD19+ B Cells in Assessing the Relapse of Neuromyelitis Optica Spectrum Disorder Patients Treated With Rituximab

**DOI:** 10.1002/brb3.70950

**Published:** 2025-10-10

**Authors:** Jing Zeng, Mingyue Zou, Mengjun Min, Min Xu

**Affiliations:** ^1^ Department of Pharmacy The Sixth Hospital of Wuhan, Affiliated Hospital of Jianghan University Wuhan China

**Keywords:** AQP4‐IgG seropositivity; CD3+CD56+ NKT cells, CD19+ B cells, neuromyelitis optica spectrum disorders, relapse, rituximab

## Abstract

**Aim:**

The purpose of this study was to explore the value of pre‐treatment levels of CD3+CD56+ NKT cells and CD19+ B cells in evaluating the relapse of patients with neuromyelitis optica spectrum disorders (NMOSD) treated with rituximab.

**Methods:**

A total of 166 NMOSD patients admitted to our hospital from January 2021 to August 2022 were included in this study. All patients received rituximab and were followed up for 1 year. Based on their relapse status, patients were categorized into a relapse group (*n* = 10) and a non‐relapse group (*n* = 156). Peripheral venous blood samples were collected, and the pre‐treatment levels of CD3+CD56+ NKT and CD19+ B cells were measured using flow cytometry. Logistic regression analysis was used to identify factors influencing relapse, and receiver operating characteristic (ROC) curve analysis was performed to evaluate the predictive value of these cells for relapse.

**Results:**

The relapse group had significantly lower levels of CD3+CD56+ NKT cells and higher levels of CD19+ B cells compared to the non‐relapse group (*p* < 0.05). Multivariate analysis confirmed that lower pre‐treatment CD3+CD56+ NKT cell levels (OR = 1.549, 95% CI: 1.068–2.199; *p* = 0.001) and higher CD19+ B cell levels (OR = 1.272, 95% CI: 1.039–1.758; *p* = 0.002) were potential independent predictors of relapse. ROC curve analysis showed that the combined model yielded a modest predictive value (AUC = 0.652, *p* < 0.05), which was statistically superior to that of either biomarker alone. During follow‐up, rituximab was generally well‐tolerated, with infusion‐related reactions (18.1%) and infections (11.4%) being the most common adverse events.

**Conclusion:**

Pre‐treatment levels of CD3+CD56+ NKT cells and CD19+ B cells are potential markers for evaluating the risk of relapse in NMOSD patients treated with rituximab, although their predictive accuracy is modest.

## Graphical abstract

This graphical abstract summarizes a study on 166 NMOSD patients treated with rituximab. Pre‐treatment assessment of peripheral CD3+CD56+ NKT cells and CD19+ B cells revealed that low NKT (≤9.85%) and high B cell (≥5.12%) levels are associated with increased relapse risk. Conversely, high NKT and/or low B cell levels correlate with lower relapse risk. Combining these biomarkers significantly improves relapse prediction (AUC = 0.619), highlighting their value in stratifying patients and guiding personalized therapeutic strategies.

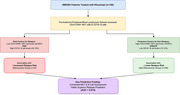

## 1 Introduction

According to a 2022 epidemiological survey, the incidence rate of neuromyelitis optica spectrum disorders (NMOSD) in China is 0.278 per 100,000 person‐years, with rates of 0.075 per 100,000 person‐years in children and 0.347 per 100,000 person‐years in adults. The incidence is notably higher in females than in males, with a ratio of 4.52:1 (Cacciaguerra and Flanagan 2024; Redenbaugh and Flanagan 2022). The observed female predominance in NMOSD is thought to be multifactorial, potentially involving: (1) hormonal influences, as sex hormones like estrogen can modulate immune responses and increase relapse risk during pregnancy/postpartum periods (Bove et al. 2017); (2) genetic predispositions, possibly linked to X‐chromosome genes (e.g., Foxp3 regulating T‐cell function) and epigenetic mechanisms (Jarius et al. 2023; Nytrova and Dolezal 2022); (3) distinct immunological pathways that enhance females' susceptibility to autoantibody‐mediated astrocytopathy, particularly in AQP4‐IgG seropositive cases (Nytrova and Dolezal 2022). NMOSD is a central nervous system inflammatory disease that primarily affects the optic nerves, spinal cord, and medulla, leading to severe outcomes such as paralysis, blindness, respiratory failure, and even death (Shi et al. 2022). The disease is characterized by rapid onset, frequent relapses, and can result in irreversible neurological damage, necessitating prompt and effective clinical intervention.

High‐dose methylprednisolone pulse therapy is the first‐line treatment for acute NMOSD, facilitating neurological recovery and reducing inflammation, yet relapse remains a significant challenge (Etemadifar et al. 2022). Rituximab, a monoclonal antibody targeting CD20, has been demonstrated in multiple studies to be effective as a maintenance therapy for NMOSD (Cruz et al. 2021). Compared to other anti‐CD20 agents like inebilizumab or ocrelizumab, rituximab's efficacy is well‐established in resource‐limited settings due to its broader accessibility (Kaegi et al. 2019). Despite this, some patients experience relapse shortly after initiating rituximab therapy or during maintenance treatment (Wingerchuk et al. 2015).

Previous studies have highlighted the role of B‐cell subpopulations, particularly CD19+ B cells and CD19/CD27 memory B cells, in mediating NMOSD pathogenesis and relapse (Fujihara et al. 2023). AQP4‐specific IgG is considered the primary pathogenic mediator in NMOSD, with over 80% of patients testing positive for AQP4‐IgG (Kaegi et al. 2019; Wingerchuk et al. 2015). Aquaporin‐4 (AQP4) is the most abundant water channel protein in the central nervous system, predominantly expressed on astrocytic end‐feet at the blood‐brain barrier and other glia–vascular interfaces (Castañeyra‐Ruiz et al. 2013; Hubbard et al. 2015; Lapshina and Ekimova 2024). Its primary function is to regulate water homeostasis and facilitate glial cell communication (Pham et al. 2024; Xiao and Hu 2014). In NMOSD, AQP4‐IgG autoantibodies bind to these AQP4 channels on astrocytes (Ratelade et al. 2013). This binding initiates a cascade of immunological events, including complement‐dependent cytotoxicity (CDC) and antibody‐dependent cellular cytotoxicity (ADCC), leading to astrocyte damage and death (astrocytopathy) (Ratelade et al. 2013; Redenbaugh and Flanagan 2022). The loss of astrocytes disrupts the blood‐brain barrier, promotes inflammatory cell infiltration (including neutrophils and eosinophils), and subsequently causes secondary demyelination and neuronal injury, characteristic of NMOSD lesions (Li et al. 2022; Redenbaugh and Flanagan 2022). Within AQP4‐IgG‐positive lesions, various immune cells, including B and T lymphocytes, are present and can induce inflammatory responses leading to necrosis and cavitation (Lucchinetti 2002). B lymphocytes are known to produce autoantibodies against AQP4 (Miao et al. 2023), which can destroy astrocytes through complement‐mediated cytotoxicity, resulting in inflammatory demyelination within the central nervous system (McCombe and Pittock 2022; Papadopoulos et al. 2014; Redenbaugh and Flanagan 2022).

CD3+CD56+ NKT cells, a subset of innate lymphocytes with immunoregulatory functions, may modulate B‐cell activity and autoantibody production (Zhao et al. 2021). Limited data are available on their role in predicting relapse in NMOSD patients treated with rituximab. This study specifically focuses on CD3+CD56+ NKT and CD19+ B cells due to their dual roles in humoral immunity (via CD19+ B cells) and immune regulation (via NKT cells), which may synergistically influence relapse risk.

## 2 Materials and Methods

### 2.1 Clinical Data

**FIGURE 1:**
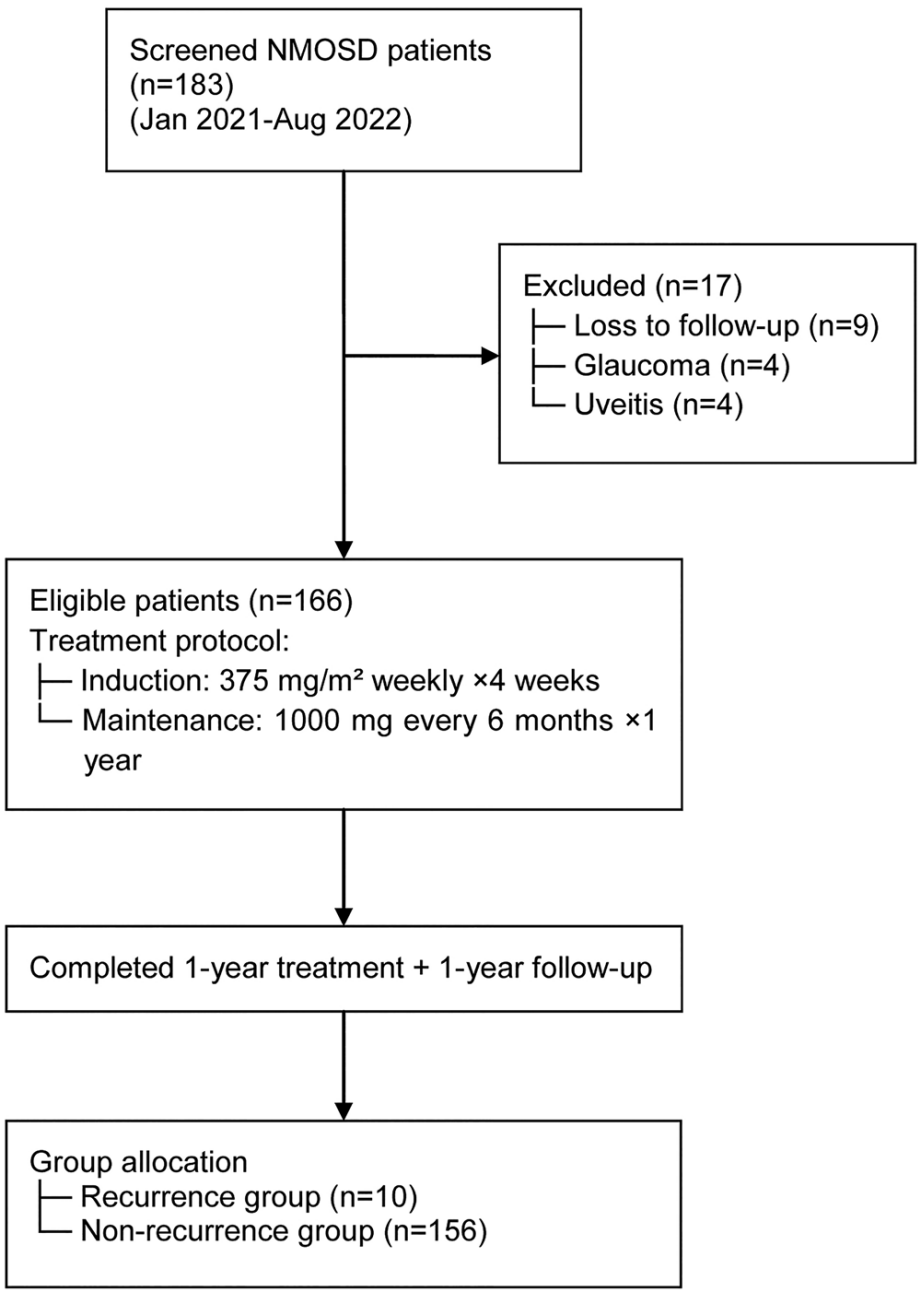
Patient enrollment flowchart.

This study included 183 patients diagnosed with neuromyelitis optica spectrum disorders (NMOSD) who were admitted to our hospital from January 2021 to August 2022. **Figure** **1** illustrates the patient enrollment process following STROBE guidelines. Initially, 183 patients were screened, of whom 17 were excluded (9 due to loss to follow‐up, 4 with glaucoma, and 4 with uveitis). The remaining 166 patients met the inclusion criteria and were enrolled. All patients were treated with rituximab for 1 year and were followed up for an additional year. Based on their relapse status, the patients were divided into two groups: the relapse group (*n* = 10) and the non‐relapse group (*n* = 156).

### 2.2 Inclusion and Exclusion Criteria

Inclusion criteria were as follows: Age at onset between 18 and 75 years (inclusive); Meeting the international consensus diagnostic criteria for NMOSD (Wingerchuk et al. 2015) in the remission phase as of 2015; AQP4‐IgG positivity confirmed via cell‐based assay (CBA, Euroimmun AG, Lübeck, Germany) in peripheral blood at the initial visit.

Exclusion criteria included: patients with severe infections, cardiovascular, cerebrovascular, liver, kidney, or hematopoietic system diseases; patients with concomitant uveitis, glaucoma, retinal diseases, or other ophthalmic conditions; patients with poor compliance who could not complete the follow‐up; patients with chronic infectious diseases such as tuberculosis or hepatitis; patients with other neurological or psychiatric disorders; patients with known allergies; pregnant patients or those planning to become pregnant; patients who were lost to follow‐up or withdrew from the study midway; patients on long‐term immunosuppressants like azathioprine, thiopurine, or cyclophosphamide; cases where non‐neurological or other neurological diseases affect the Expanded Disability Status Scale (EDSS) score.

### 2.3 Treatment Methods

Patients initially received an intravenous infusion of methylprednisolone sodium succinate (manufactured by China Resources Zongsheng Pharmaceutical Co., Ltd., China, approval number H20040844, 250 mg per vial) at a dose of 1000 mg/day for 3 days. The dosage was then halved every 3 days until reaching 125 mg/day. Subsequently, patients were switched to oral methylprednisolone tablets (produced by Zhejiang Xianjie Pharmaceutical Co., Ltd., China, approval number H20213809, 16 mg per tablet) at a dose of 48 mg/day, with a weekly reduction of 4 mg until reaching a maintenance dose of 4 mg/day or discontinuation.

All patients were treated with a rituximab induction‐maintenance protocol (375 mg/m^2^ weekly for 4 weeks as induction, followed by 1000 mg every 6 months as maintenance therapy) for 1 year. This therapeutic strategy, combining an initial B‐cell depletion phase with subsequent maintenance infusions, is a widely adopted approach for long‐term management in NMOSD, although specific regimens may vary (Clarke et al. 2020).

#### 2.3.1 Concurrent Use of Infliximab

Infliximab (100 mg weekly for 4 weeks) was administered to 51 patients with co‐existing AQP4‐IgG and MOG‐IgG positivity to address potential refractory inflammation (Zhao et al. 2022). To mitigate potential adverse reactions, 20 mg of promethazine was injected intramuscularly, and 1 tablet of ibuprofen was administered orally 30 min before the infliximab injection. In the latter half of the year, patients received a reduced dose of infliximab injection solution (50 mg) twice.

#### 2.3.2 Rituximab Retreatment Criteria

Retreatment was initiated if CD19+ B cells exceeded 1% of total lymphocytes or upon clinical relapse.

### 2.4 Observation Indicators

Clinical data collected from the two groups included age, gender, and disease course, among other variables.

#### 2.4.1 Flow Cytometry Analysis

Peripheral blood samples were collected before treatment. CD3+CD56+ NKT and CD19+ B cells were quantified using a BD FACSCanto II flow cytometer (BD Biosciences, USA). Lymphocytes were gated using forward/side scatter, followed by CD3‐FITC/CD56‐PE and CD19‐APC staining (BioLegend, USA).

#### 2.4.2 EDSS Assessment

EDSS scores were described as mean ± standard deviation and analyzed using the *t*‐test.

#### 2.4.3 Statistical Adjustments

Baseline confounders (prior immunosuppressants, disease duration) were included as covariates in regression models.

### 2.5 Statistical Methods

Statistical analysis was conducted using SPSS version 26.0. Normality of continuous data was assessed using Shapiro–Wilk or Kolmogorov–Smirnov tests, alongside visual inspection of histograms and Q–Q plots. Continuous data following a normal distribution were described as mean ± standard deviation and analyzed using the *t*‐test. For data not following a normal distribution, the median and interquartile range (IQR) were used, and the Mann–Whitney *U* test was applied. Categorical data were described as frequencies (percentages). The Wilcoxon test was used for comparing ordinal data, while the *χ* ^2^ test was applied for unordered categorical data. Due to the small number of events (*n* = 10 in the relapse group) and the imbalanced group sizes, multivariate logistic regression with Firth's bias reduction was employed to minimize small‐sample bias. Firth's method was chosen as it provides a robust, penalized‐likelihood‐based solution that reduces bias and produces finite estimates even in cases of data separation, which can occur with rare events, making it preferable to standard logistic regression in this context (Heinze and Schemper 2002). Receiver operating characteristic (ROC) curve analysis was used to assess the predictive value of CD3+CD56+ NKT and CD19+ B cells for relapse.

## 3 Results

### 3.1 Comparison of Clinical Data Between Groups

A comprehensive comparison was conducted between the relapse group (*n* = 10) and the non‐relapse group (*n* = 156) across various clinical parameters. No significant differences were observed between the two groups in terms of gender (*p* = 0.648), disease duration (4.18 ± 1.07 vs. 4.09 ± 1.12 years, *p* = 0.805), initial site of involvement (*p* = 0.949), number of attacks (*p* = 0.088), EDSS score (4.98 ± 1.52 vs. 4.84 ± 1.42, *p* = 0.764), MOG antibody positivity (3/10 vs. 48/156, *p* = 0.944), MRI findings of the involved site (general categories, *p* = 0.955), CSF IgA (*p* = 0.180), CSF IgM levels (*p* = 0.193), white blood cell count in the cerebrospinal fluid (CSF) (*p* = 0.104), or CSF albumin levels (*p* = 0.084). All patients were AQP4‐IgG‐positive by inclusion criteria. Statistically significant differences were found in age (46.29 ± 10.27 vs. 38.93 ± 10.09 years, *p* = 0.027), autoimmune antibody positivity (8/10 vs. 79/156, *p* = 0.047; see Table 1 footnote), spinal cord segment involvement on MRI (6.14 ± 1.02 vs. 5.17 ± 1.18 segments, *p* = 0.012), CSF IgG levels (13.76 ± 1.93 vs. 12.41 ± 1.82 µmol/L, *p* = 0.025), and CSF oligoclonal band (OCB) positivity (4/10 vs. 0/156, *p* < 0.001) (**Table** **1**). These results indicate that older patients, those with more extensive spinal cord damage evident on MRI, higher CSF IgG levels, the presence of OCBs in the CSF, and positivity for other autoimmune antibodies were more likely to be in the relapse group.

**TABLE 1:** Comparison of clinical data between the relapse group (*n* = 10) and the non‐relapse group (*n* = 156).

Indicator	Relapse group (*n* = 10)	Non‐relapse group (*n* = 156)	*t*/*χ* ^2^	*p*‐value
Age (years)	46.29 ± 10.27	38.93 ± 10.09	−2.234	0.027
Gender (*n*)			0.208	0.648
Male	3	58		
Female	7	98		
Disease duration (years)	4.18 ± 1.07	4.09 ± 1.12	−0.247	0.805
Initial involved sites (*n*)			0.721	0.949
Optic nerve	5	67		
Spinal cord	2	51		
Brainstem	1	14		
Thalamus	1	12		
Mixed involved sites	1	12		
Number of relapses	2.53 ± 0.56	2.19 ± 0.61	−1.716	0.088
EDSS score	4.98 ± 1.52	4.84 ± 1.42	−0.301	0.764
MOG antibody positivity (*n*)	3	48	0.005	0.944
Autoimmune antibody positivity (*n*)	8	79	3.963	0.047
MRI indicated involved sites (*n*)			0.093	0.955
Spinal cord	5	83		
Brain	3	40		
Optic nerve	2	33		
MRI indicated segments affected in spinal cord (*n*)	6.14 ± 1.02	5.17 ± 1.18	−2.538	0.012
CSF white blood cells (×10⁹/L)	5.39 ± 0.76	5.01 ± 0.71	−1.634	0.104
CSF albumin (g/L)	0.59 ± 0.13	0.50 ± 0.16	−1.741	0.084
CSF immunoglobulins (µmol/L)				
IgA	2.38 ± 0.51	2.11 ± 0.62	−1.347	0.180
IgM	1.31 ± 0.27	1.13 ± 0.43	−1.306	0.193
IgG	13.76 ± 1.93	12.41 ± 1.82	−2.266	0.025
CSF OCB positivity (*n*)	4	0	66.343	<0.001

*Note*: Autoimmune antibody positivity: defined as positivity for anti‐SSA/SSB, anti‐thyroid peroxidase (anti‐TPO), or anti‐nuclear antibodies (ANA). Co‐positivity: all patients were AQP4‐IgG‐positive; MOG‐IgG co‐positivity occurred in 3/10 (30%) relapse and 48/156 (30.8%) non‐relapse cases (*p* = 0.944).

### 3.2 Comparison of CD3+CD56+ and CD19+ B Levels Between Groups

**FIGURE 2:**
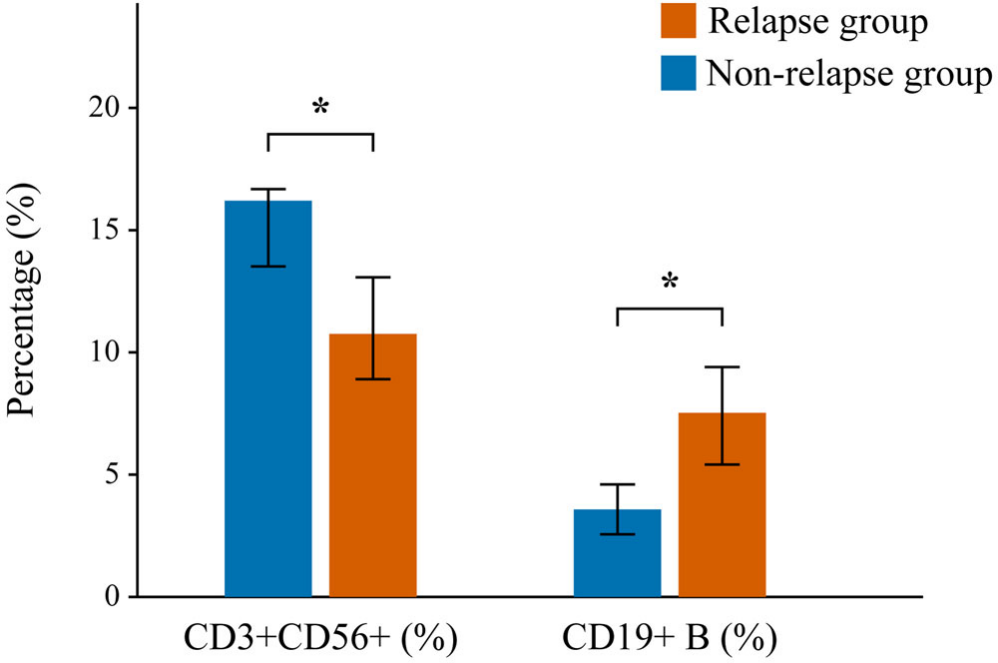
Comparison of pre‐treatment CD3+CD56+ NKT and CD19+ B cell levels between groups. The bar chart displays the mean percentage of CD3+CD56+ NKT cells and CD19+ B cells in the relapse group (*n* = 10) and the non‐relapse group (*n* = 156). Compared to the non‐relapse group, patients in the relapse group showed significantly lower levels of CD3+CD56+ NKT cells and significantly higher levels of CD19+ B cells. Error bars represent standard deviation (SD). ***p < 0.001.

Pre‐treatment CD3+CD56+ NKT cell percentages were significantly lower in the relapse group (10.76 ± 2.31% vs. 13.92 ± 2.76%, *p* < 0.001), whereas CD19+ B cell percentages were higher (7.53 ± 1.87% vs. 3.58 ± 1.02%, *p* < 0.001) (Table 2 and Figure 2). These findings suggest that, prior to treatment, patients who later experienced relapse exhibited a relative deficiency in immunoregulatory NKT cells and a higher burden of antibody‐producing B cells, potentially predisposing them to a less favorable response to rituximab. This direct comparison in **Figure** **2** visually underscores the association between these baseline immune cell profiles and the subsequent clinical outcome of relapse versus non‐relapse.

**TABLE 2:** Comparison of CD3+CD56+ and CD19+ B levels (mean ± SD) between the relapse and non‐relapse groups.

Indicator	Relapse group (*n* = 10)	Non‐relapse group (*n* = 156)	*T*	*p*‐value
CD3+CD56+ (%)	10.76 ± 2.31	13.92 ± 2.76	3.539	<0.001
CD19+ B (%)	7.53 ± 1.87	3.58 ± 1.02	−11.170	<0.001

### 3.3 Univariate Logistic Regression Analysis of Factors Influencing Relapse

Univariate analysis identified age (OR = 2.857, 95% CI: 1.692–3.879, *p* < 0.001), spinal cord segment involvement on MRI (OR = 7.976, 95% CI: 3.005–18.757, *p* < 0.001), lower CD3+CD56+ NKT cells (OR = 1.549, 95% CI: 1.068–2.199, *p* = 0.001), and higher CD19+ B cells (OR = 1.272, 95% CI: 1.039–1.758, *p* = 0.002) as significant predictors of relapse (**Table** **3**). This suggests that older age, greater extent of spinal cord lesions, lower NKT cell levels, and higher B cell levels were individually associated with an increased likelihood of relapse.

**TABLE 3:** Univariate logistic regression analysis of factors influencing the relapse of NMOSD patients treated with rituximab.

Indicator	*β*	SE	Wald *χ* ^2^	OR (95% CI)	*p*‐value
Age	1.039	0.253	17.547	2.857 (1.692–3.879)	<0.001
MRI showing segmental involvement of spinal cord	2.156	0.487	16.287	7.976 (3.005–18.757)	<0.001
Cerebrospinal fluid immunoglobulin	0.097	0.165	0.336	0.916 (0.073–1.324)	0.566
IgG	0.082	0.238	0.316	1.082 (0.676–1.716)	0.736
Cerebrospinal fluid OCB positivity	2.046	1.123	3.216	3.287 (0.861–7.765)	0.069
CD3+CD56+	0.568	0.189	6.529	1.549 (1.068–2.199)	0.001
CD19+ B	0.287	0.103	7.639	1.272 (1.039–1.758)	0.002

### 3.4 Multivariate Logistic Regression Analysis of Factors Influencing Relapse

In multivariate analysis, CD3+CD56+ NKT cells (OR = 1.549, 95% CI: 1.068–2.199, *p* = 0.001) and CD19+ B cells (OR = 1.272, 95% CI: 1.039–1.758, *p* = 0.002) remained potential independent predictors, while age and spinal cord involvement lost significance (**Table** **4**). This suggests that even after accounting for other factors, lower pre‐treatment CD3+CD56+ NKT cell levels and higher pre‐treatment CD19+ B cell levels may be independently associated with an increased risk of relapse in NMOSD patients following rituximab therapy.

**TABLE 4:** Multivariate logistic regression analysis of factors influencing the relapse of NMOSD in patients treated with rituximab.

Indicator	*β*	SE	Wald *χ* ^2^	OR (95% CI)	*p*‐value
Age	0.788	0.647	1.487	2.207 (0.761–6.373)	0.219
MRI showing segmental involvement of spinal cord	0.082	0.238	0.316	1.082 (0.676–1.716)	0.736
CD3+CD56+	0.438	0.145	9.124	1.549 (1.068–2.199)	0.001
CD19+ B	0.241	0.087	7.639	1.272 (1.039–1.758)	0.002

### 3.5 Evaluation of Predictive Value for Relapse Based on CD3+CD56+ and CD19+ B Levels

**FIGURE 3:**
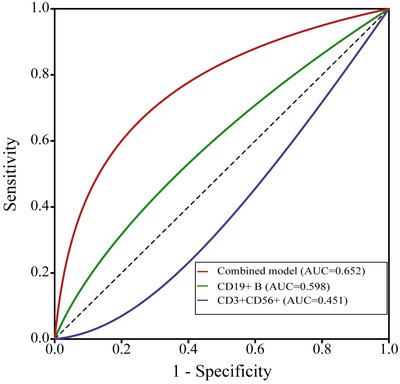
Receiver operating characteristic (ROC) curve analysis for predicting relapse. The ROC curves illustrate the predictive value of pre‐treatment CD3+CD56+ NKT cells (AUC = 0.451), CD19+ B cells (AUC = 0.598), and a combined model for predicting relapse in NMOSD patients treated with rituximab. The combined assessment yielded a modest but statistically superior predictive value compared to either marker alone (AUC = 0.652). The diagonal line represents a random guess (AUC = 0.5).

**FIGURE 4:**
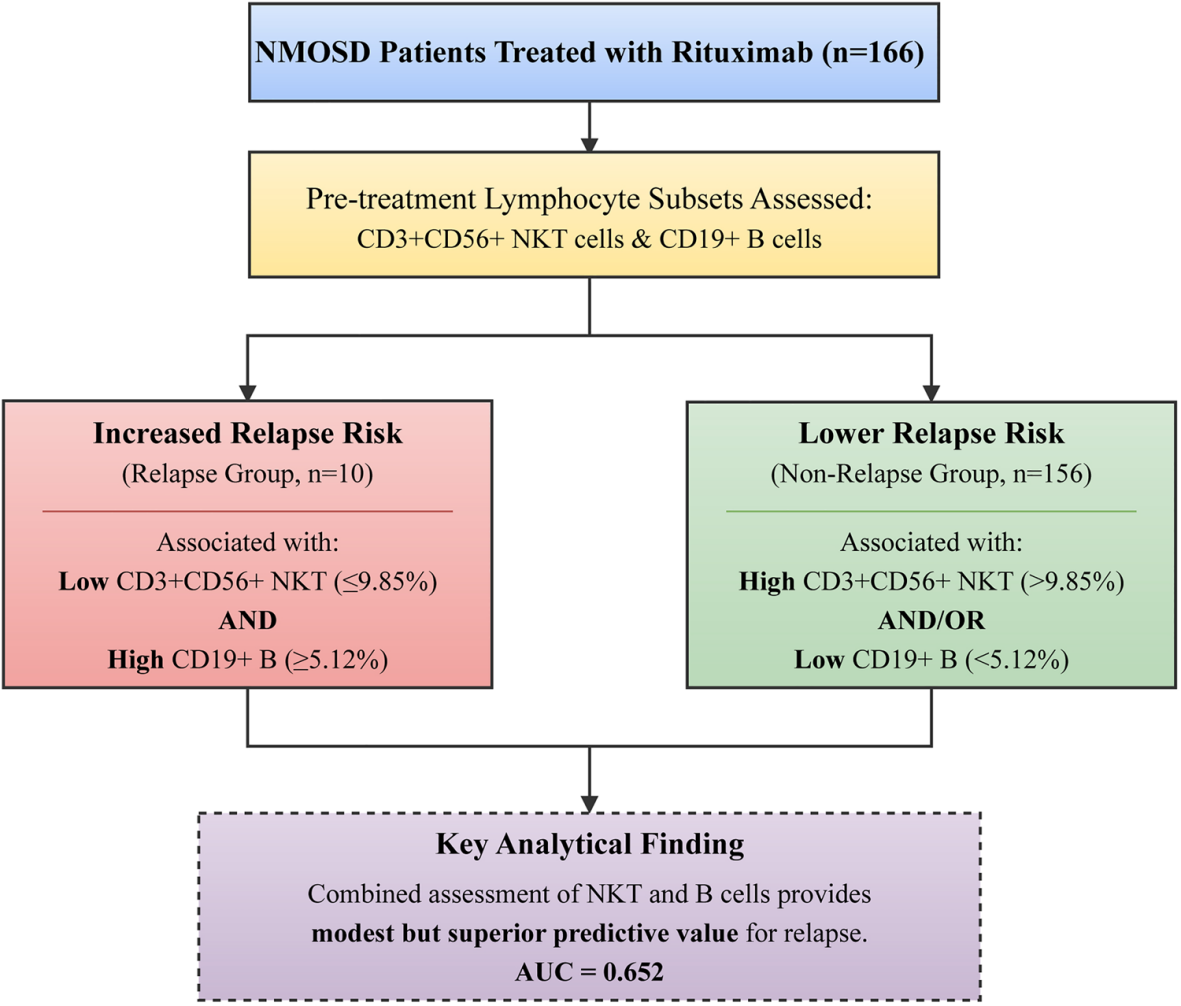
Conceptual diagram illustrating the relationship between pre‐treatment CD3+CD56+ NKT and CD19+ B cell levels and the risk of relapse in NMOSD patients treated with rituximab. Lower NKT cell levels and higher B cell levels are associated with an increased risk of relapse. The combined assessment of these markers provided modest but statistically superior predictive value (AUC = 0.652).

ROC analysis revealed that the combined model (CD3+CD56+ + CD19+ B) had the highest diagnostic accuracy (AUC =  0.652, sensitivity =  90.0%, specificity =  80.1%), with optimal cut‐off values of ≤9.85% for CD3+CD56+ and ≥5.12% for CD19+ B (Table 5 and Figure 3). This analysis suggests that combining these markers provides a statistically superior, albeit modest, assessment of relapse risk in NMOSD patients undergoing rituximab therapy. However, with an AUC of 0.652, the model's discriminative ability is considered poor to fair and highlights the need for additional predictive markers. To visually summarize the relationship between baseline lymphocyte subsets and relapse, **Figure** **4** illustrates how lower pre‐treatment CD3+CD56+ NKT cell levels and higher pre‐treatment CD19+ B cell levels are associated with an increased risk of relapse in NMOSD patients treated with rituximab. The figure also highlights the superior predictive value of the combined assessment of these markers.

**TABLE 5:** Evaluation of CD3+CD56+ and CD19+ B levels in predicting NMOSD relapse in patients treated with rituximab.

Indicator	AUC (95% CI)	Sensitivity	Specificity	Optimal cut‐off
CD3+CD56+ (%)	0.451 (0.289–0.613)	80.0%	73.1%	≤9.85%
CD19+ B (%)	0.598 (0.435–0.761)	80.0%	71.8%	≥5.12%
Combined model	0.652 (0.510–0.794)	90.0%	80.1%	—

### 3.6 Safety and Tolerability of Rituximab

During the 1‐year treatment and 1‐year follow‐up period, rituximab was generally well‐tolerated. The most common adverse events were infusion‐related reactions and infections. Infusion‐related reactions occurred in 30 (18.1%) patients, were typically mild to moderate (Grade 1–2), and managed with temporary infusion interruption and symptomatic treatment. Infections were reported in 19 (11.4%) patients, with urinary tract infections (*n* = 8) and upper respiratory tract infections (*n* = 7) being the most frequent. All infections were successfully treated with standard antibiotics. Four (2.4%) patients developed mild, transient neutropenia that resolved without intervention. No adverse events led to the permanent discontinuation of the treatment. A detailed summary of adverse events is provided in Table **6**.

**TABLE 6:** Summary of adverse events in the study cohort (*n* = 166).

Adverse event category	Event type	Number of patients, *n* (%)
**Infusion‐related reactions (total)**	Mild (e.g., pruritus, rash)	22 (13.3)
	Moderate (e.g., fever, chills, hypotension)	8 (4.8)
	**Overall**	**30 (18.1)**
**Infections (total)**	Upper respiratory tract infection	7 (4.2)
	Urinary tract infection	8 (4.8)
	Other (e.g., cellulitis, gastroenteritis)	4 (2.4)
	**Overall**	**19 (11.4)**
**Hematologic abnormalities**	Transient neutropenia (Grade 1–2)	4 (2.4)

## 4 Discussion

Neuromyelitis optica spectrum disorders (NMOSD) are a group of central nervous system demyelinating diseases primarily driven by abnormal immune function, with humoral immunity playing a critical role in their pathogenesis. Due to the high relapse rate associated with NMOSD, patients often require long‐term immunosuppressive therapy, which not only imposes a significant financial burden but also leads to adverse effects on liver, kidney, and gastrointestinal functions (Xue et al. 2023). Therefore, early identification of factors that contribute to relapse is essential for optimizing therapeutic interventions and controlling disease progression.

Rituximab, a CD20 monoclonal antibody, has been widely adopted in the treatment of autoimmune diseases, including NMOSD. Although its efficacy and safety in NMOSD have been well established, some patients still experience relapses shortly after treatment (Barreras et al. 2022). Compared to newer B‐cell depletion therapies such as inebilizumab (anti‐CD19) and satralizumab (anti‐IL‐6 receptor), rituximab's broader B‐cell targeting may contribute to variability in relapse prevention (Kaegi et al. 2019). Rituximab was first administered to eight patients with NMOSD at the University of California, San Francisco, Multiple Sclerosis Center in 2005. It was observed that rituximab achieved a high rate of B lymphocyte depletion, which could be sustained for 6–12 months following induction therapy. During a follow‐up period of 6–18 months, six patients remained relapse‐free, while two experienced mild myelitis relapses, yielding a relapse rate of 25% (Cree et al. 2005). Subsequent studies by Barreras et al. (2022) indicated that most AQP4‐IgG positive NMOSD patients are prone to relapse within the first 6 months of rituximab treatment or when rituximab infusion is delayed. In our study of 166 NMOSD patients treated with rituximab, 10 experienced relapse, resulting in a relapse rate of 6.02%, which is lower than rates reported with inebilizumab (12%–15%) or satralizumab (20%–30%) (Kim et al. 2015). This lower rate may reflect concurrent use of methylprednisolone or other immunosuppressants in our cohort, which were not systematically analyzed here.

Our findings revealed that age, the number of spinal cord segments involved as detected by MRI, autoimmune antibody positivity, serum IgG levels, and cerebrospinal fluid oligoclonal band (OCB) positivity differed significantly between the relapse and non‐relapse groups (*p* < 0.05). Notably, AQP4‐IgG/MOG‐IgG co‐positivity (3/10 vs. 48/156, *p* = 0.944) did not influence relapse risk, suggesting biomarker validity is preserved regardless of serostatus. These results are consistent with the findings of Hu et al. (2022) and Maillart et al. (2020), who also reported that older age, a higher number of spinal cord segments involved, elevated IgG levels, and cerebrospinal fluid OCB positivity are associated with a higher risk of relapse in NMOSD patients.

Rituximab therapy in NMOSD is typically divided into two phases. In the first phase, almost all CD19+ and CD20+ cells are depleted, disrupting T‐B cell interactions and sharply reducing the number of antibody‐producing plasma cells. In the second phase, B cells begin to reappear. Riva et al. (2023) found that CD19+ B cells were completely suppressed 1 month after rituximab treatment (range 0%–0.1%), with this suppression lasting for at least 5 months. However, by the sixth month, CD19+ B cells began to increase again. Therefore, monitoring peripheral blood lymphocyte subpopulations during rituximab treatment is crucial for understanding the factors that trigger relapse in NMOSD.

Natural killer T (NKT) cells are a distinct subset of T lymphocytes with characteristics of both NK cells and T lymphocytes. While most studies suggest that NKT cells play significant roles in autoimmune regulation, bacterial infection resistance (e.g., Mycobacterium), and anti‐tumor immunity (Nishiyama et al. 2022; Yao et al. 2024), research on CD3+CD56+ NKT cells—a key subset of CD3+ T lymphocytes—remains limited in the context of NMOSD (Yang et al. 2022). Moreover, during rituximab treatment, NKG2C‐positive NK cells within the NKT cell population may enhance antibody‐dependent cell‐mediated cytotoxicity, leading to increased B cell depletion. Our study found that CD3+CD56+ levels were lower in the relapse group, whereas CD19+ B levels were higher (*p* < 0.05), suggesting that patients who relapse have reduced CD3+CD56+ levels and elevated CD19+ B levels. The reduced NK lymphocyte levels, particularly NKG2C+ NK lymphocytes, in relapsing NMOSD patients may impair antibody‐dependent cell‐mediated cytotoxicity, thereby diminishing B cell depletion (Moreira et al. 2022). However, the exact mechanisms require further investigation.

Additionally, Yandamuri et al. (2020) reported that the decrease in CD16+CD56+ NK lymphocytes in NMOSD patients treated with rituximab was not significantly different from those not treated with rituximab, suggesting that the regulatory mechanisms of NKT cells may vary depending on the disease state. Different immunosuppressive agents may affect NKT cell function through distinct pathways, and the role of NKT cells in immune monitoring warrants further exploration.

This study highlights that the factors influencing relapse in NMOSD patients treated with rituximab include age, the number of spinal cord segments involved, CD3+CD56+ levels, and CD19+ B levels. Multivariate logistic regression analysis identified CD3+CD56+ and CD19+B levels as potential independent factors associated with relapse, possibly related to incomplete B lymphocyte depletion or proliferation. Our findings are in line with previous research suggesting the clinical utility of these lymphocyte subsets. For example, a study by Khani et al. (2022) suggested that CD3+CD56+ cell counts can be used to evaluate autoimmune diseases, including MS and NMOSD, while work by Levy and Mealy (2021) indicated that CD19+ B cells are closely associated with NMOSD prognosis. Furthermore, Cao et al. (2023) found that monitoring CD19+ B cells is useful for predicting relapse in NMOSD patients undergoing low‐dose rituximab treatment.

ROC curve analysis in this study demonstrated that the combined diagnosis using CD3+CD56+ and CD19+ B levels yielded a statistically higher AUC for predicting relapse in NMOSD patients treated with rituximab, compared to the use of either marker alone (*p* < 0.05). This suggests that combining these markers provides a more accurate, albeit modest, assessment of relapse risk in NMOSD patients undergoing rituximab therapy. However, this study has limitations: (1) The small relapse group (*n* = 10) and the highly imbalanced group sizes significantly limit statistical power and increase the risk of type II errors. This imbalance might question the robustness of our findings, and the results should be interpreted as preliminary. This small sample size for the relapse group could also lead to an overestimation of effect sizes. (2) There is potential for patient selection bias, as this was a single‐center study, and patients included might not be fully representative of the broader NMOSD population. For instance, patients with very severe or rapidly progressing disease might be managed differently or referred to specialized centers, potentially skewing the cohort towards a particular disease spectrum. (3) Variations in individual treatment response to rituximab, which can be influenced by unmeasured genetic factors, disease heterogeneity, or previous treatment history, were not exhaustively explored. Although we aimed to control for some confounders, residual confounding variables affecting the results cannot be entirely ruled out. (4) MRI lesion location details (e.g., specific brain regions beyond general categories) and systemic complement activation levels, which could provide further prognostic insights, were not analyzed in detail. (5) The predictive accuracy of our combined model, while statistically significant, was modest (AUC = 0.652). An AUC in this range is generally considered to have poor to fair discriminative ability, indicating that these biomarkers alone are insufficient for definitive clinical decision‐making and should be considered exploratory. (6) Although we reported key adverse events, our safety data collection was not exhaustive and lacked systematic grading of severity for all event types. Future studies should aim for larger, multi‐center cohorts with longer follow‐up periods, include longitudinal tracking of these and other biomarkers (e.g., different B‐cell subsets, cytokine profiles), validate findings in MOG antibody‐associated disease (MOGAD) cohorts, and potentially compare these biomarkers' predictive utility against those for newer biologic therapies.

In summary, pre‐treatment CD3+CD56+ and CD19+ B levels exhibit abnormal expression patterns in NMOSD patients who relapse after rituximab treatment. These markers may be influential factors in relapse and could potentially be utilized to assess the likelihood of relapse in NMOSD patients treated with rituximab.

## Author Contributions

JZ and MZ contributed to the conception and design of the study. MM contributed to the acquisition of data. MX contributed to the analysis of data. JZ and MZ wrote the manuscript. MM and MX supervised the revision of the manuscript. MM wrote the final manuscript. All authors approved the final version of the manuscript.

## Ethics Statement

The study protocol was approved by the Ethics Committee of The Sixth Hospital of Wuhan (NO.24‐23A), Affiliated Hospital of Jianghan University, and the study was performed in accordance with the Helsinki II declaration. Informed consent was obtained from all the study subjects before enrollment.

## Conflicts of Interest

The authors declare that they have no competing interests.

## Peer Review

The peer review history for this article is available at https://publons.com/publon/10.1002/brb3.70950.

## Data Availability

Data are provided within the manuscript files.
